# A novel approach: Simulating multiple simultaneous encounters to assess multitasking ability in emergency medicine

**DOI:** 10.1371/journal.pone.0257887

**Published:** 2021-09-28

**Authors:** Wen-Cheng Huang, Shih-Chang Hsu, Chih-Hao Yang, Che-Wei Lin, Fat-Moon Suk, Kai-Chun Hu, Yun-Yu Wu, Hao-Yu Chen, Chin-Wang Hsu

**Affiliations:** 1 Department of Emergency, School of Medicine, College of Medicine, Taipei Medical University, Taipei, Taiwan; 2 Emergency Department, Department of Emergency and Critical Medicine, Wan Fang Hospital, Taipei Medical University, Taipei, Taiwan; 3 Center for Education in Medical Simulation, Taipei Medical University, Taipei, Taiwan; 4 Department of Education and Humanities in Medicine, School of Medicine, College of Medicine, Taipei Medical University, Taipei, Taiwan; 5 Division of Gastroenterology, Department of Internal Medicine, Wan Fang Hospital, Taipei Medical University, Taipei, Taiwan; 6 Department of Internal Medicine, School of Medicine, College of Medicine, Taipei Medical University, Taipei, Taiwan; Harper University Hospital, UNITED STATES

## Abstract

**Study objective:**

The purpose of this feasibility study is to develop and validate a new assessment tool and scoring system for multitasking competency for physicians in-training in a timed simulated setting. The multitasking competency includes ability to appropriately prioritize and implement tasks for different patients who present simultaneously.

**Methods:**

We designed three single task stations with different levels of difficulty and priority. These skill stations were then combined to create a multitasking simulation scenario. Skill checklists and the global rating scale were utilized to assess the participants’ performance. A multitasking score, multitasking index, and priority score were developed to measure the multitasking ability of participants.

**Results:**

Thirty-three first-year postgraduate physicians were recruited for this prospective study. The total performance scores were significantly higher for the single-tasking stations than for the multitasking scenario. In terms of the time needed to complete the tasks, the participants spent more time on the multitasking scenario than on the single-tasking scenario. There were significant correlations between the global rating scale and the multitasking score (rho = 0.693, p < 0.001) and between the global rating scale and the multitasking index (rho = 0.515, p < 0.001). The multitasking score, multitasking index, and priority score did not have any significant correlations with the total single-tasking score.

**Conclusion:**

We demonstrated that the use of a simulated multitasking scenario could be an effective method of assessing multitasking ability and allow assessors to offer better quality feedback.

## Introduction

In the current healthcare system, physicians’ abilities to manage frequent interruptions and prioritize essential tasks in a timely manner have become increasingly valuable [1]. Especially in an emergency department setting, interruptions are part of the working conditions. The high frequency of interruptions experienced during a shift by emergency medicine (EM) physicians has been documented [2, 3]. Previous studies demonstrated that interruptions divert physicians’ attention away from their primary tasks. Interruptions that lead to breaks in a task could cause medical errors and increase the potential for harming patients [4, 5]. In an ideal environment, distractions would be minimized or eliminated; however, distractions are inevitable in the emergency room setting. Therefore, the EM physician must learn how to cope in a multitasking environment.

The ability to multitask has been recognized as an essential skill in emergency medicine [6]. In EM, multitasking is defined as the ability to prioritize and implement the evaluation and management of multiple patients in the emergency department, including handling interruptions and task switching to provide optimal care [6–8]. The Accreditation Council for Graduate Medical Education (ACGME) and the American Board of Emergency Medicine developed “The Emergency Medicine Milestone Project,” which provides a framework for the assessment of the development of the resident physician in key dimensions of the elements of physician competency in the specialty [9]. Multitasking is one of the sub competencies in patient care. The current method of evaluating multitasking ability is mainly dependent on direct observation. The mini-clinical examination (mini-CEX) and the standardized direct observation tool for EM (SDOT) are the two most commonly used direct observation tools [6, 10, 11]. Although simulation-based assessment is one of the evaluation methods suggested by The Emergency Medicine Milestone Project, such a method’s validity has not yet been documented.

While medical simulation has become a standard educational modality within graduate and undergraduate medical education, there are few publications regarding the use of simulation as an evaluation tool in patient care [12–16]. A few studies have demonstrated using simulations of multiple encounters to teach multitasking [17–19]. However, utilizing multiple encounter simulation-based methods to assess multitasking skills has not been fully investigated. The purpose of this feasibility study is to develop and validate a new assessment tool and scoring system for multitasking competency for physicians in-training in a timed simulated setting.

## Methods

In this study, we designed a simulated multitasking scenario which included three skills (intubation, bedside ultrasonography and suturing) stations with different levels of priority and difficulty. In the scenario, participants performed these tasks simultaneously, and assign priorities according to their perception. The main objective of this study design is to validate the feasibility of this tool to measure the ability to multitask. We further observed whether the novel method could assess learners’ performance and provide educators additional information to provide feedback.

### Study population

This study enrolled first-year postgraduate physicians who had 2 years of clinical experience and completed one-month emergency and simulation training at an academic center in Taiwan. Participation in this study was voluntary and no remuneration was provided. A total of 33 first year post-graduates were recruited via email and social media.

### Study design

We designed three short single task stations with different levels of difficulty and priority (low, moderate, high). Intubation tasks were considered as high priority, bedside ultrasonography was moderate and wound suturing was low priority.

These three short single-task stations were combined to create a multitask scenario.

Participants completed three independent single task scenarios and one combined multitasking scenario, and were evaluated using a standardized checklist for each task. The checklist for the multitasking scenario was a combination of the checklists of the single task scenarios. We compared the score of the 3 single task stations against the score of each station in the multitasking scenario to determine the impact of multitasking on skill performance. To reduce the testing effect bias, approximately half of the participants (n = 16) were assessed on the multitasking scenario first. The remaining participants completed the single-tasking stations first. Participants were randomized to either the multitasking simulation first or skill stations in series first. A computer-generated list of random numbers was used for the allocation.

### Ethical approval

This observational and prospective study was approved by the Association of Taipei Medical University Joint Institutional Review Board (TMU-JIRB NO: N201808021).

We explained the purpose and methods (The participants were videotaped and the videos were reviewed by raters) of the research in written documents and regarded the return of completed survey forms as consent to participate in the research.

### Scenario design

**Fig 1:**
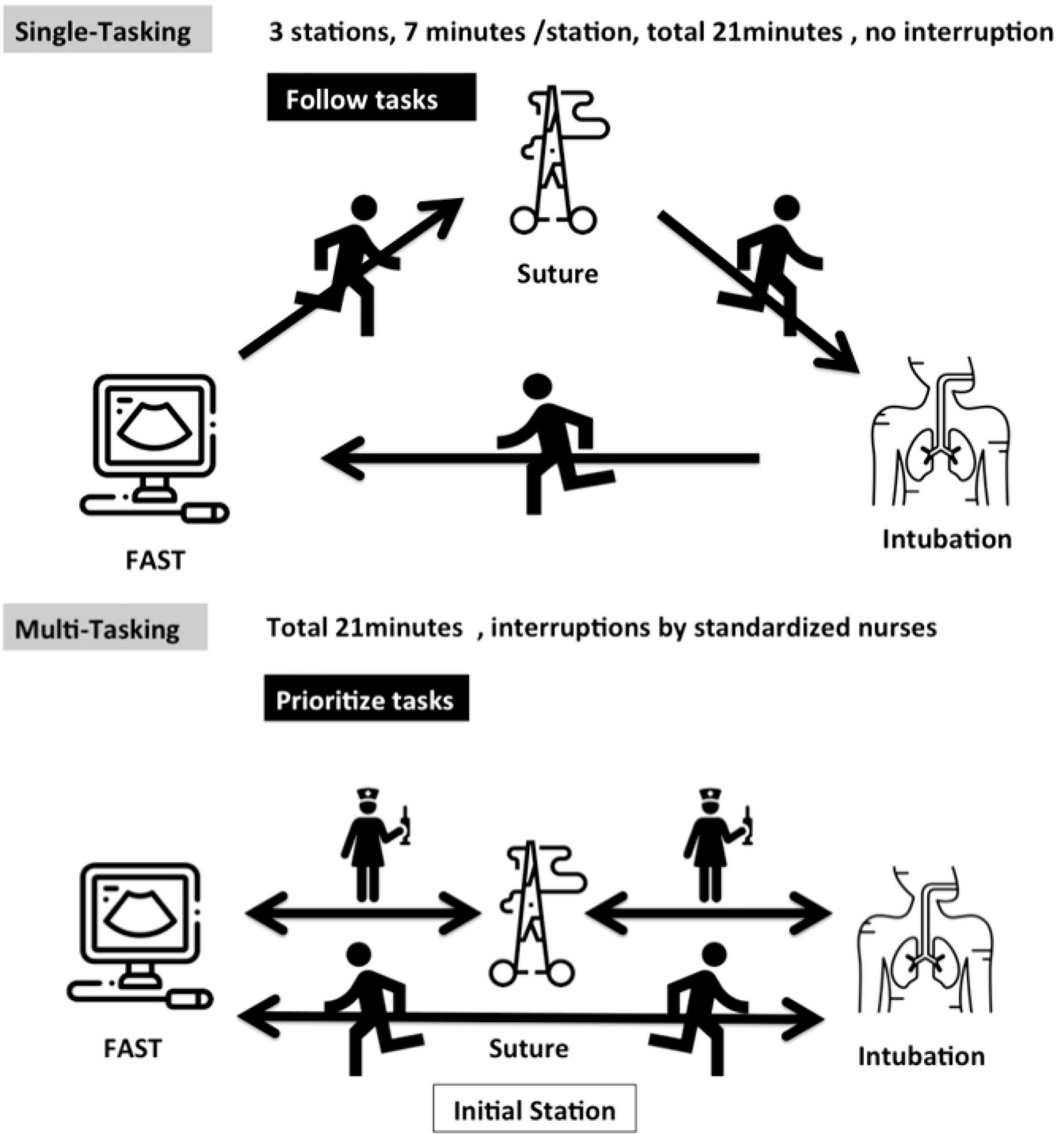
Process and scenario descriptions for the single-tasking and multitasking evaluations.

The three skill stations and their priority and difficulty levels are summarized in Table 1. The completion time for each single task station was recorded. In the multitasking scenario, we individually recorded the total amount of time spent at each of the 3 tasks. In the multitasking evaluation, all participants started at the suture station (low priority). During the scenario, two standardized nurses delivered updates about the other stations at preset times (e.g., intubation station: poor oxygen saturation with respiratory failure; ultrasound station: severe abdominal pain with hypotension). The participants had to make decisions about which task to handle first. While working on any given station, they faced two interruptions with status updates from the other stations (Fig 1, S1 File).

**Table 1:** Details of the three scenarios with different priorities.

Station 1	Station 2	Station 3
**Suture Station**	**Ultrasound station**	**Intubation station**
Low Priority	Moderate priority	High priority
40-year-old man with forearm laceration 3 cm	30-year-old man with blunt abdominal trauma	78-year-old man with respiratory failure
**Simulation modality:** Part-Task Trainer with an assistant (nurse)	**Simulation modality**: Standardized patient	**Simulation modality**: High fidelity Manikin with an assistant (nurse)
**Task**: suture with 3 stitch and wound cover	**Task**: perform focus assessment with sonography for trauma (FAST)	**Task**: perform intubation and explain the need of intubation to family

### Scoring method and measurement

**Fig 2:**
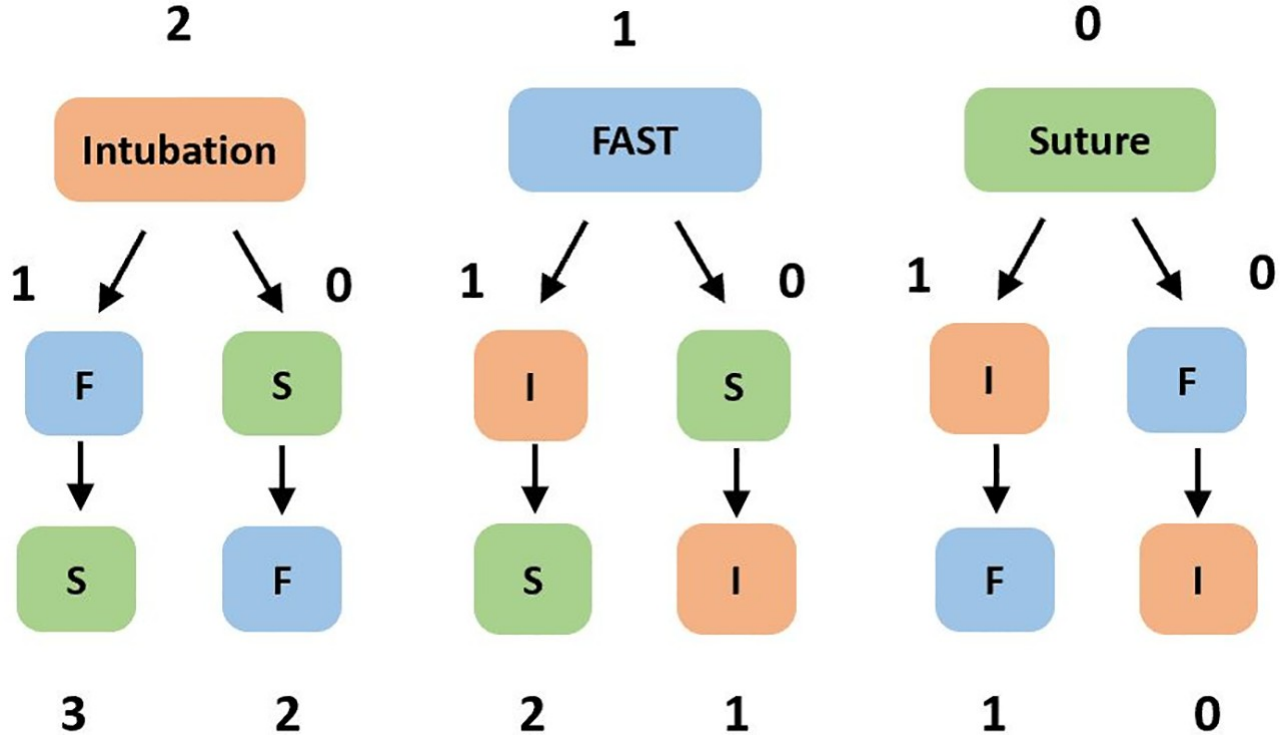
The algorithm used to calculate the priority score. (I: intubation; F: FAST; S: suture).

Each scenario was assessed by two experienced (over ten years of practice) emergency physicians. Each single task scenario was evaluated using a standardized checklist previously validated by the clinical and educational experts’ consensus and analysis in Taiwan. The multitasking scenario was evaluated using the same checklists (S1 Table) and an additional Global Rating Scale (GRS) based on the 14th sub competency (Multitasking) of the Emergency Medicine Milestones (S2 File) [23]. A priority score was also determined according to the order in which the participant completed the tasks. To measure multitasking ability in the combined simulation scenario, we used a multitasking score (MS), multitasking index (MTI) and a priority score (PS). The MS is the sum of the individual task checklist scores to assess the ability of task completion in the multitasking scenario. In addition, the MTI was defined as the multitasking simulation score divided by the sum of the single-tasking simulation scores. The MTI is designed to quantify the ratio of task completion and build a model to directly observe multitasking performance. To correctly assess the multitasking ability of the participants, they must have an acceptable level of competency in each of the skills included. As a result, the MTI scores were calculated only for the participants who scored greater than 50% on all three single-tasking stations. Multitasking ability includes the performance of each independent skill and the ability to prioritize the performance of each of the scenario tasks. Consequently, we developed the algorithm shown in Fig 2 to calculate the PS. The participants’ performances were scored by raters using the above tools as well as the GRS for the single-tasking stations and the multitasking scenario.

### Scenario rating

All raters had completed standardized training and held a valid certification as OSCE raters by the Taiwan Medical Education Association. To assess inter-rater reliability, 12 additional participants were recruited and assessed in 3 single-task and a multitasking scenario. All scenarios were assessed by a single assessor and video recorded. The video recording was assessed by a second assessor with no previous knowledge of the initial assessment score. (The inter-rater reliability for all encounters is provided in S3 File)

### Statistical analysis

The statistical analysis of the data obtained in the study was performed with R 3.2.4 software (R Foundation for Statistical Computing, Vienna, Austria). Continuous variables are expressed as the means and standard deviations (SDs) or medians and interquartile ranges. Categorical variables are expressed as counts or percentages. The Mann-Whitney U-test was used for continuous variables that did not adhere to a parametric distribution. The degree of association between variables was measured by the Spearman rank correlation test. All statistical tests were two-sided and paired. A value of p < 0.05 was considered statistically significant.

## Results

**Fig 3:**
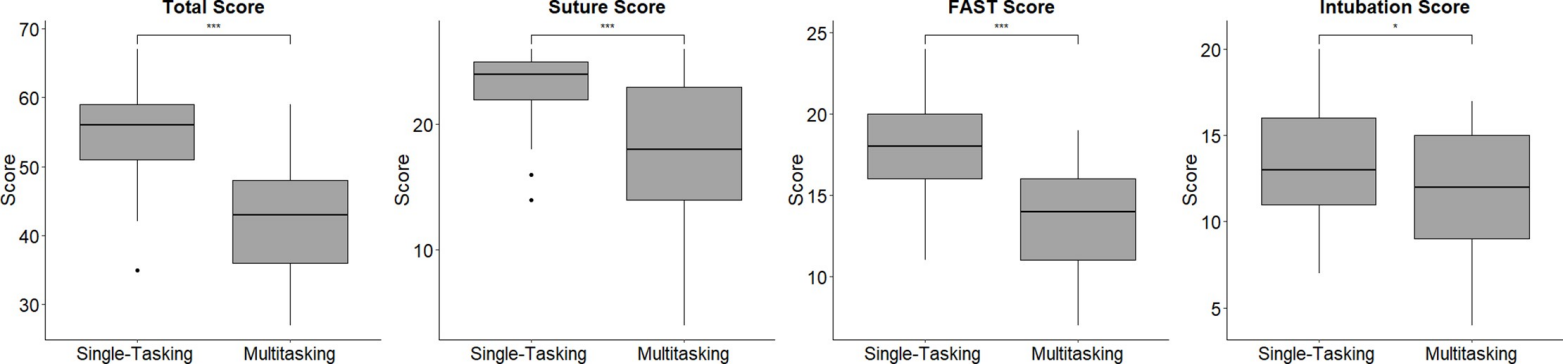
The distribution of the total scores on the single-tasking and multitasking scenarios (single-tasking: median = 56, IQR = 8; multitasking: median = 43, IQR = 12; ***p <0.0001) and the distribution of individual scores on the single-tasking and multitasking scenarios (single-tasking suture score: median = 24, IQR = 3; multitasking suture score: median = 18, IQR = 9; single-tasking FAST score: median = 18, IQR = 4; multitasking FAST score: median = 14, IQR = 5; single-tasking intubation score: median = 13, IQR = 5; multitasking intubation score: median = 12, IQR = 6; ***p <0.0001, * p<0.05).

**Fig 4:**
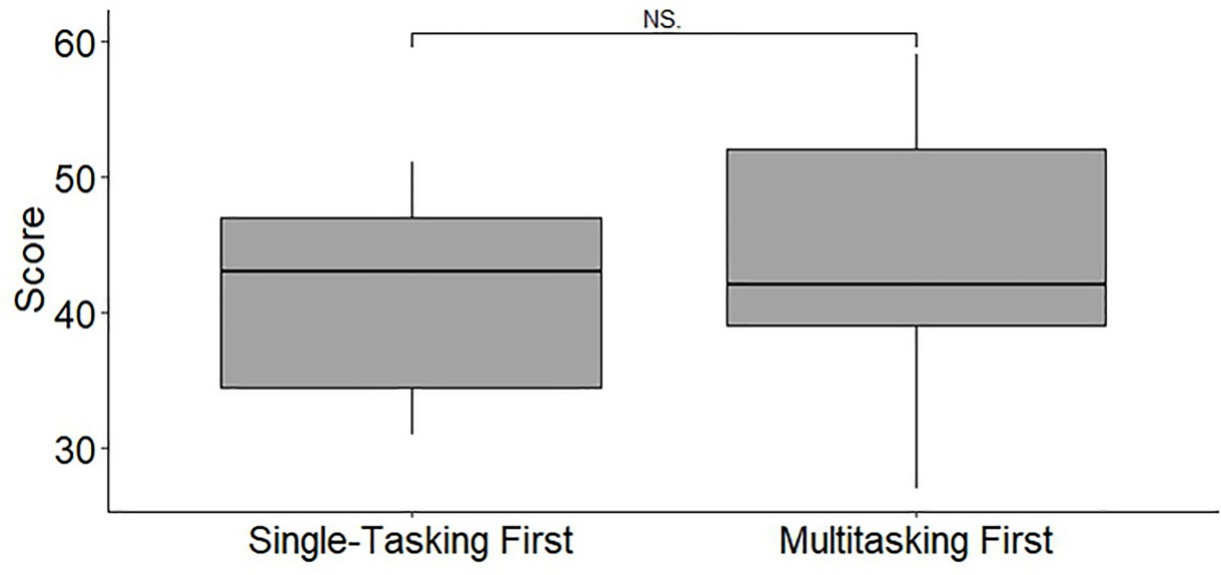
The distribution of total multitasking scores in the participants who were first evaluated on the single-tasking scenarios or the multitasking scenario (single-tasking first: median = 43, IQR = 12.5; multitasking first: median = 42, IQR = 13; NS: no significant).

**Fig 5:**
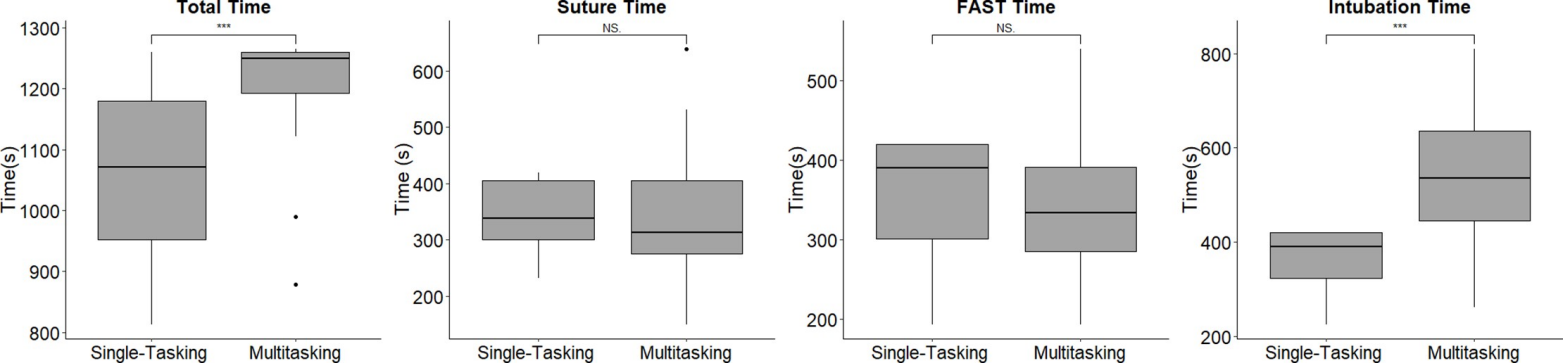
The distribution of the total completion times for the single-tasking and multitasking scenarios (single-tasking: median = 1071, IQR = 227; multitasking: median = 1250, IQR = 67; *** p <0.0001) and the distribution of the individual completion times for the single-tasking and multitasking scenarios (single-tasking suture score: median = 338, IQR = 105; multitasking suture score: median = 313, IQR = 129; single-tasking FAST score: median = 390, IQR = 119; multitasking FAST score: median = 334, IQR = 106; single-tasking intubation score: median = 390, IQR = 97; multitasking intubation score: median = 535, IQR = 190; *** p <0.0001, NS: nonsignificant).

The baseline characteristics of the participants are described in Table 2. In total, 33 first-year medical graduates were included in this study, of whom 20 (60.61%) were male. The median age of the medical graduates was 25 years (IQR: 25 years to 25 years). The total performance scores were significantly higher for the single-tasking scenarios than for the multitasking scenario. After stratifying the scores by different priorities, the scores at each station were still significantly higher for the single-tasking scenarios than for the multitasking scenario (Fig 3). Additionally, the order in which the scenarios were evaluated did not influence the MS (Fig 4). In terms of the time needed to complete the tasks, the participants spent more time on the multitasking scenario than on the single-tasking scenarios. However, the subgroup data suggested that the participants used a significant amount of time on intubation in the multitasking scenario (Fig 5). To assess the prioritization ability of the participants, the PS was used. The median of the PS was 3 (IQR = 3–2). There were significant correlations between the GRS and the MS (rho = 0.693, p < 0.001). In our data of MTI, 6 participants who do not satisfy the criteria were excluded from the calculations (the participants need to obtain greater than 50% of score on each single-tasking station). There were also significant correlations between the GRS and the MTI (rho = 0515, p < 0.001). However, the GRS was not significantly correlated with the PS. The MS, MTI, and PS were not significantly correlated with the total single-tasking score (Table 3).

**Table 2:** The characteristics of the participants.

Characteristic	Median (IQR) or Frequency (%)
**Age (years), median (IQR)**	25.15 (0)
**Male, n (%)**	20 (60.61)
**Total participants, n (%)**	33 (100)

**Table 3:** The correlations between the global rating scale score, multitasking score, multitasking index and total single-tasking score.

Variable	Multitasking Score		Multitasking Index		Priority scores	
	Spearman rho	p value	Spearman rho	p value	Spearman rho	p value
**Global Rating Scale**	0.693	< 0.001*	0.515	< 0.001*	0.314	0.074
**Total Single-Tasking Score**	-0.333	0.058	-0.257	0.149	0.102	0.572

* Significant (p value <0.05).

## Discussion

The ability to multitask demonstrates two core competencies, namely, **prioritization** when faced with multiple patients and **implementation** to maintain focus on individual patients [20–22]. In the past, the methods used to evaluate multitasking ability were based on direct observation by clinical preceptors [10, 23]. However, these methods require a relatively long period for observation. Additionally, multitasking situations in clinical practice are often unpredictable and uncontrollable.

The accurate measurement of multitasking ability requires specific clinical challenges to be present simultaneously. In this study, we have demonstrated the use of a simulated multitasking scenario with tasks with different priority levels to assess physicians’ multitasking abilities.

In this study, we observed that the clinical performance of the participant was affected by multi patient encounters. In single-tasking scenarios, participants can concentrate on a single task; there is no prioritization needed, and less psychological stress is imposed. When faced with the need for multitasking, participants experience interruptions. In addition, they are required to prioritize patients, which imposes more psychological stress. Our results indicate that the overall performance scores for the single-tasking stations were higher than those for the multitasking scenario.

The results correspond with the findings of A Parush et al. which proposed that the multitasking environment affects EM physicians’ concentration and performance [24].

When comparing the participants’ performance on the multitasking scenario and each single-tasking station, Our results showed that in the multitasking scenario, participants allocated significantly more time on the intubation station than on the lower priority stations (suture/FAST). This result can be explained by the findings of a previous publication, which suggests that participants prioritize the more critical patients and spend more time completing the tasks associated with those patients [25]. Conversely, they spend less time on the other stations with lower priority levels, and these results are also consistent with real clinical practice [26]. During multitasking situations, in high-priority tasks the performance of the clinicians was relatively unaffected but they required additional time to complete these tasks. Low-priority tasks were easily neglected in the multitasking setting. This finding reveals that it is important to provide additional training or clinical assistance to address this issue.

Our study showed a significant positive correlation between the MTI and the traditional GRS score of direct observation in the multitasking scenario.

If MTI is used for the assessment of multitasking ability, the efficacy will be similar to direct observation, indicating that we could use the multitasking scenario to assess participants’ multitasking ability under safe and controllable simulated conditions. The MS and GRS scores were also positively correlated. However, we believed that the Multitasking Index could better represent the participant’s implementation ability in a multitasking setting since it considers the participant’s single-tasking score (Their baseline ability). The MTI could be used to establish a standard tool to assess the competencies of different levels of physicians.

In addition, we also used the PS to evaluate the decision-making abilities of the participants during the multitasking evaluation. Interestingly, the PS was not correlated with the GRS score. This may be because the GRS focuses primarily on the completion of the clinical tasks and does not emphasize decision making. Prioritization is a crucial skill in clinical practice, especially during multiple patient encounters. In the past, we emphasized the completion of clinical tasks when evaluating multitasking, but prioritization is an important core competency and component of multitasking [27]. Therefore, we believe that the PS should remain a reference parameter when evaluating a participant’s multitasking ability.

In previous studies assessing multitasking, direct observation was the major technique used [10, 23]. For EM residents, multitasking ability is commonly assessed with the Mini-CEX and SDOT in clinical settings. However, evaluating multitasking in an emergency room can cause clinical interference and assess performance less objectively [28]. It is impossible to have enough suitable patients for clinical preceptors to be able to evaluate multitasking at any time in clinical practice. Therefore, the well-designed multiple encounter simulation developed for this study can be used to observe targeted clinical competencies. Moreover, this method of the assessment does not interfere with clinical work and preserves patient safety. Additionally, multitasking simulation can provide comparable assessment accuracy to the traditional forms of assessment by direct observation.

### Limitations

Some limitations of this study should be noted. As this was a pilot study, only 33 participants, carried out at a single institution. The sample size and the single institution may limit result generalizability. Because we only enrolled first-year medical graduates, gold standards for multitasking could not be established. Future studies, should include larger sample sizes and participants at different levels of professional development, such as EM residents and EM attending physicians. In addition, we hope to identify specific factors that negatively impact performance during multi patient encounters. A better understanding of these factors and their impact on task performance will allow the development of better training methods or courses. Finally, the checklists that were utilized in our study only assessed basic competency, but provide limited information about task performance quality.

## Conclusion

Using simulated multi-patient encounters, this study shows initial validity that the multitasking core competency can be assessed using three newly developed assessment tools: the multitasking score, the multitasking index, and the priority score. This setting may allow educators to offer concrete and timely feedback to trainees without interference to emergency patient care.

## Supporting information

S1 File The multitasking scenario assessment demonstration. (DOCX)

S2 File Global Rating Scales for multitasking scenario. (DOCX)

S3 File The inter-rater reliability for all encounters assessed. (DOCX)

S1 Table Checklists for stations and multitasking scenario. (DOCX)
