# Supplementary material for: A novel approach: Simulating multiple simultaneous encounters to assess multitasking ability in emergency medicine
Source: PLoS One. 2021 Sep 28;16(9):e0257887. doi: 10.1371/journal.pone.0257887 (PMC8478191; doi:10.1371/journal.pone.0257887)
Supplement: S1 File — (DOCX) [file pone.0257887.s001.docx]

**S1 Video:** The Multitasking Scenario Assessment Demonstration

YouTube on line: <https://www.youtube.com/watch?v=ig_jcXA8EKo&feature=youtu.be>
