# Supplementary figures and images for: A novel approach: Simulating multiple simultaneous encounters to assess multitasking ability in emergency medicine
Source: PLoS One. 2021 Sep 28;16(9):e0257887. doi: 10.1371/journal.pone.0257887 (PMC8478191; doi:10.1371/journal.pone.0257887)

**S3 Figure: Global Rating Scales for multitasking scenario**


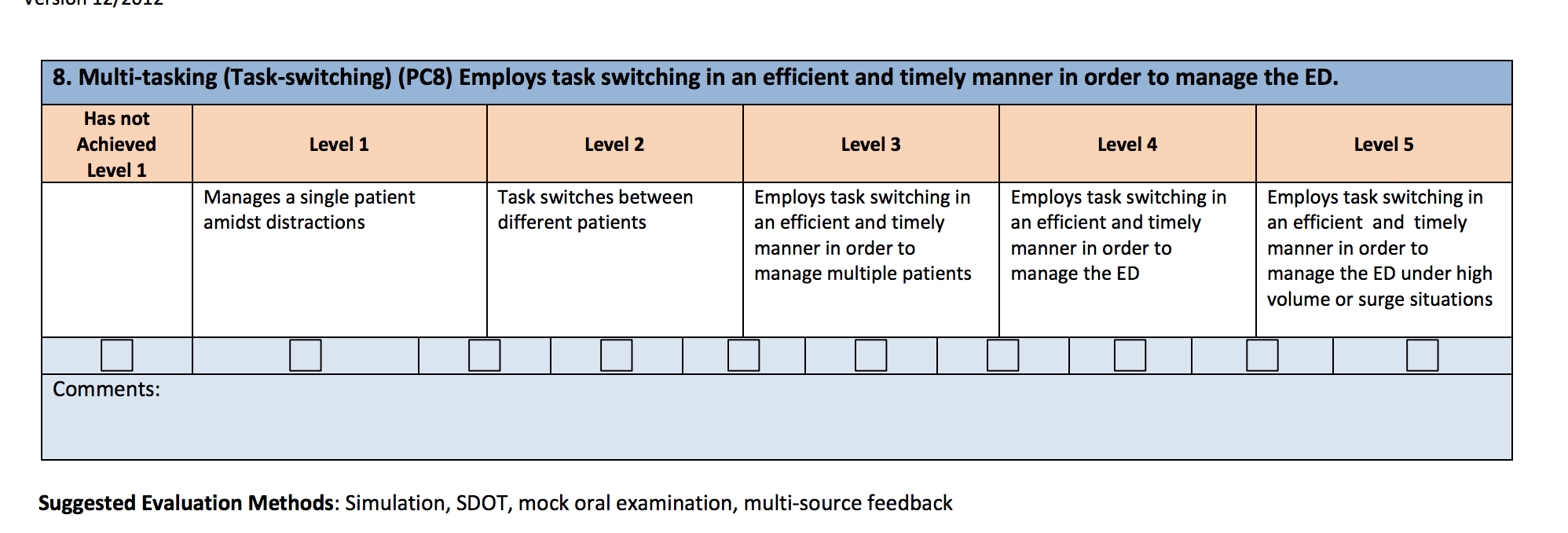

Supplement: S2 File — (DOCX) [file pone.0257887.s002.docx]
