# Supplementary material for: A novel approach: Simulating multiple simultaneous encounters to assess multitasking ability in emergency medicine
Source: PLoS One. 2021 Sep 28;16(9):e0257887. doi: 10.1371/journal.pone.0257887 (PMC8478191; doi:10.1371/journal.pone.0257887)
Supplement: S3 File — (DOCX) [file pone.0257887.s003.docx]

**S4 File: The inter-rater reliability for all encounters assessed**

The Kappa values of the scenarios were as follows:

Single Task Assessments:

Suturing Station Cohen’s Kappa (unweighted) = 0.81, z= 7.75, p-value = 8.88e-15

FAST station Cohen’s Kappa (unweighted) = 0.788, z = 5.87, p-value = 4.43e-09

Intubation Scenario Cohen’s Kappa (unweighted) = 0.808, z = 7.36, p-value = 1.78e-13

Multitasking Assessments:

PRS Cohen’s Kappa (unweighted) = 0.859, z = 4.48, p-value = 7.63e-06

IRS Cohen’s Kappa (unweighted) = 1, z = 5.51, p-value = 3.58e-08
