# Supplementary material for: A novel approach: Simulating multiple simultaneous encounters to assess multitasking ability in emergency medicine
Source: PLoS One. 2021 Sep 28;16(9):e0257887. doi: 10.1371/journal.pone.0257887 (PMC8478191; doi:10.1371/journal.pone.0257887)
Supplement: S1 Table — (DOCX) [file pone.0257887.s004.docx]

**S2 Table: Checklists for stations and multitasking scenario**

FAST Station

| Item | Scores  0    1   2   note | | | |
| --- | --- | --- | --- | --- |
| Introduce themselves and identify the patient |  |  |  |  |
| Explained the indication for performing FAST (suspect internal bleeding) |  |  |  |  |
| Exposed the scanning area |  |  |  |  |
| Stood at the right side of the patient |  |  |  |  |
| Scanned Morrison’s pouch correctly |  |  |  |  |
| Scanned Splenorenal recess correctly |  |  |  |  |
| Scanned Douglas pouch correctly |  |  |  |  |
| Scanned pericardial space correctly |  |  |  |  |
| Identified free fluid in Morrison’s pouch correctly |  |  |  |  |
| Explained the possibility of internal bleeding |  |  |  |  |
| Explained the disposition and arrange a CT scan for diagnosis |  |  |  |  |
| Noticed the discomfort of patient and provided pain control |  |  |  |  |

Intubation Station

| Item | Scores  0     1   2    note | | | |
| --- | --- | --- | --- | --- |
| Explained the indication of intubation |  |  |  |  |
| Explained the contraindication of intubation |  |  |  |  |
| Explained the process of intubation |  |  |  |  |
| Wore protective equipment (gloves, mask, glass) |  |  |  |  |
| Checked the intubation equipment (balloon, styles, Laryngoscope) |  |  |  |  |
| Performed preoxygenation (BVM ventilation) |  |  |  |  |
| Performed correct steps of intubation |  |  |  |  |
| Requested assistance when needed |  |  |  |  |
| Correct placement of Endotracheal tube (location/chest raise) |  |  |  |  |
| Checked the intubation location |  |  |  |  |
| Arranged X-ray for confirmation |  |  |  |  |

Suture Station

| Item | Scores  0    1 2 note | | | |
| --- | --- | --- | --- | --- |
| Chose appropriate needle type |  |  |  |  |
| Wore Sterile Gloves correctly |  |  |  |  |
| Used needle holder correctly 1. Clamp 1/3 of needle tail |  |  |  |  |
| Used needle holder correctly 2. No needle loss |  |  |  |  |
| Used needle holder correctly 3. No repeated needling |  |  |  |  |
| Used Tips correctly |  |  |  |  |
| Performed suture correctly (3 tight knots every stitch) 1^st^ tie |  |  |  |  |
| Performed suture correctly (3 tight knots every stitch) 2^nd^ tie |  |  |  |  |
| Performed suture correctly (3 tight knots every stitch) 3^rd^ tie |  |  |  |  |
| Kept an appropriate interval between stitches |  |  |  |  |
| Maintained aseptic technique during the whole procedure |  |  |  |  |
| Disposed of the needle correctly after finishing suture |  |  |  |  |
| Desposed of medical waste correctly after finishing suture |  |  |  |  |
